# Case report of a patient with VEXAS syndrome

**DOI:** 10.1097/MD.0000000000036738

**Published:** 2023-12-29

**Authors:** Ioannis Tsourveloudis, Eleni C. Georgiadi, Georgios Vatalis, Paraskevi Kotsi

**Affiliations:** a Transfusion Department, University General Hospital of Larissa, Larissa, Greece; b Rehabilitationszentrum, Bad Aussee Österreich, Austria.

**Keywords:** autoimmunity, myelodysplastic syndrome, UBA1 somatic mutation, VEXAS

## Abstract

**Rationale:**

Hematological malignancies have always been a challenge for scientists because there is a constant need to better define these entities. Myelodysplastic syndromes (MDS) are clonal hematopoietic disorders characterized by ineffective hematopoiesis. Cytogenetics and molecular findings are a prerequisite for these syndromes as they confirm the clonal nature of the disease. However, MDS is often linked to autoimmunity and inflammation as part of its pathogenesis. Recently, VEXAS syndrome (vacuoles, E1 enzyme, X-linked, autoinflammatory, somatic) linked these two in a single mutation, suggesting that the heterogeneity among hematological malignancies often demands a more personalized medicine by tailoring medical treatment to the individual characteristics of each patient.

**Patient concerns:**

We present a case of VEXAS syndrome regarding a 63-year-old male patient who initially presented with episodes of low fever, polyarthritis of the knees and ankles, polymyalgia, and fatigue. His laboratory examinations revealed increased levels of serum inflammatory markers.

**Diagnoses:**

Diagnosis was based on high clinical suspicion, laboratory findings, and vacuolization of the erythroid and myeloid precursors in the bone marrow evaluation. Mutational status of ubiquitin-like modifier activating enzyme 1 gene was positive with a 68.8% allelomorph frequency (rs782416867).

**Interventions:**

Therapy was based on controlling inflammation with the use of glucocorticoids and treating MDS-related anemia with the use of erythropoietin.

**Outcomes:**

Currently, the patient visits our department regularly. He is still receiving the aforementioned treatment. He did not mention any new incidents for the time being.

**Lessons:**

VEXAS syndrome as a newly identified entity might be often underestimated since its clinical presentation is notably diverse.

## 1. Introduction

Hematological malignancies have always been a challenge for scientists because of the constant need to clarify these entities. Through better understanding of the clinical aspects as well as of the molecular pathways implicated in the pathogenesis of these diseases, we can tailor targeted therapies to achieve at least prolonged progression-free survival if not remission.^[1,2]^

According to the World Health Organization, myelodysplastic syndromes (MDS) are clonal hematopoietic disorders characterized by ineffective hematopoiesis that include both morphologic dysplasia in hematopoietic cells and bone marrow failure, refractory peripheral cytopenias, and a risk of progression to acute myeloid leukemia.^[3]^ Cytogenetics and molecular findings are a prerequisite for these syndromes as they confirm the clonal nature of the disease.^[4]^ However, in hematological malignancies, not only neoplastic cells matter but also the environment of the neoplastic cell population.^[5]^ MDS is often associated with autoimmunity and inflammation to such an extent that MDS and autoimmune disorders may be 2 sides of the same coin.^[6]^ Dysregulation of the immunological environment and impaired levels of the inflammatory cytokines, such as tumor necrosis factor-α, interferon-γ, interleukin (IL)-6, IL-8, and IL-17 both seem to contribute, to some point, to the pathogenesis of the disease.^[7]^

Until recently, a novelty in the MDS landscape appeared and confirmed that MDS and autoimmunity can both be attributed to a single somatic mutation.^[8]^ VEXAS syndrome (defined as **V**acuoles, **E**1 enzyme, **X**-linked, **A**utoinflammatory, **S**omatic) is a myeloid-driven, adult-onset inflammatory syndrome associated with hematological neoplasms. The disease presents with a wide range of manifestations, including macrocytic anemia, fever, constitutional symptoms, neutrophilic dermatoses, cutaneous vasculitis, and pulmonary infiltrates, making the diagnosis challenging. A characteristic finding in the bone marrow is vacuolization of myeloid and erythroid progenitors.^[9]^

From this viewpoint, we present a case of VEXAS syndrome that is currently being treated in our department and we review the literature regarding this entity.

## 2. Case presentation

A 63-year-old male patient initially presented to his physician in 2019 with episodes of low fever, polyarthritis of the knees and ankles, polymyalgia, and fatigue. Laboratory evaluation revealed normocytic anemia, elevated erythrocyte sedimentation rate, C-reactive protein, and polyclonal hypergammaglobulinemia. Repeated detailed serological evaluations for systemic autoimmune disorders and infections were unremarkable, including negative rheumatoid factor, antinuclear antibodies, anti-proteinase 3/MPO antibodies, and normal levels of C3 and C4. The hemoglobin electrophoresis results were normal. Imaging (whole body computed tomographies) showed no evidence of solid malignancies, whereas biopsy of the left temporal artery (due to clinical suspicion) revealed no temporal arteritis.

The patient’s personal history included prolonged (>2 years) known anemia and leukopenia that had never been fully investigated in the past and subacute thyroiditis for which he received oral corticosteroids (methylprednisolone).

At that time, the patient developed several episodes of chondritis affecting the nose and ear, episcleritis, and periorbital erythema while he mentioned losing 7 to 8 kg in a month period. Due to a positive Leishmania polymerase chain reaction result, the patient received liposomal Amphotericin B (based on the protocol: dose 3 mg/kg for 5 days, total dose 21 mg/kg for days 1 to 5, d14 and d21). He was also treated periodically with corticosteroids based on constitutional symptoms and clinical improvement.

For almost 2 years, the patient was followed up with clinical evaluation and laboratory examinations without specific treatment except for corticosteroids for brief periods of time. Leukopenia was a constant finding while anemia evolved from normocytic to macrocytic (Table 1). In 2021, due to anemia, a total body computed tomography scan was repeated revealing pulmonary infiltrates and a complete hematological work up was performed (peripheral smear evaluation, bone marrow aspirate, bone marrow biopsy, Karyotype, next generation sequence). Mediterranean fever was ruled out due to negative results for the MEFV gene (mutations E148Q, P369S, F479L, M680I [G/C], M680I [G/A], I692del, M694V, M694I, K695R, V726A, A744S, and R761H).

**Table 1:** Blood indices over the years.

Blood indices	Year						
	2017	2018	2019	2020	2021	2022	2023
Hb	12.9	12.3	10.8	9,7	9,4	10	11,1
Hct	38.2	35.8	32	29	27	29.8	31.9
MCV	89	91	94	103	111	111	111
WBC	2600	2500	3700	3100	4000	6800	5000

Hb = hemoglobin, Hct = hematocrit, MCV = mean corpuscular volume, WBC = white blood cell count.

**Figure 1.**
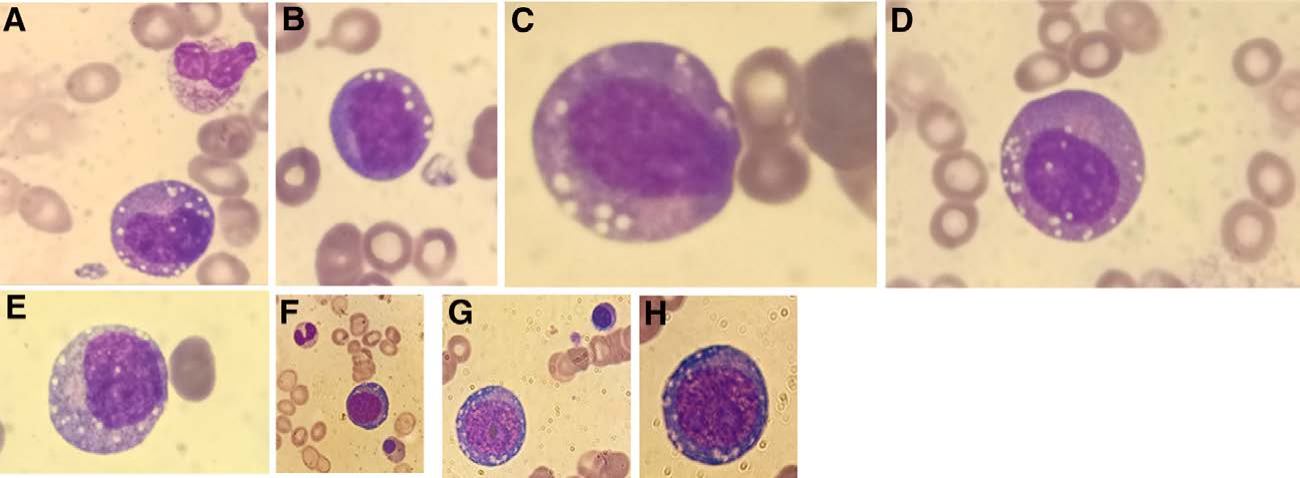
Bone marrow vacuolization in VEXAS case. Photos (a–h) illustrate bone marrow aspirate smear findings. Representative aberrant vacuolized bone marrow cells. Cytoplasmic vacuolation of granular and erythroid progenitor cells is noticed (×100 magnification).

Peripheral smear evaluation showed anisocytosis, macrocytosis, mild rouleaux, and a left shift in the presence of promyelocytes and myelocytes. Bone marrow aspirate and biopsy suggested a diagnosis of MDS with a normal karyotype. However, aberrant vacuolized bone marrow cells were observed (Fig. 1) with vacuoles appearing in myeloid lineages but not in lymphoid lineages. Moreover, it was also distinguishing that the vacuoles were not found in the cytoplasm of mature circulating blood cells. Subsequently, a clinical suspicion of VEXAS syndrome was suspected. The mutational status of the ubiquitin-like modifier activating enzyme 1 (UBA1) gene was positive with a 68.8% allelomorph frequency (rs782416867). A diagnosis of VEXAS syndrome was made and the patient was started on methylprednisolone 16 mg daily and subcutaneous erythropoietin 40,000 IU weekly.

Currently, the patient visits our department regularly for checkup. The patient is still receiving treatment with corticosteroids and erythropoietin. He did not mention any new incidents for the time being. His latest laboratory findings are shown on Table 1.

## 3. Discussion

Here we present the case of a patient with VEXAS syndrome who is currently being treated in our department.

He initially presented with constitutional symptoms and increased levels of serum inflammatory markers such as C-reactive protein and erythrocyte sedimentation rate. He subsequently developed relapsing ear and nose chondritis as well as ocular inflammation. His hematological parameters at disease onset were affected suggesting normocytic anemia that worsened over time resulting in gradual macrocytic features. A diagnosis of VEXAS syndrome was made due to high clinical suspicion, laboratory findings, and vacuolization of the erythroid and myeloid precursors in the bone marrow evaluation.

Therapy was based on controlling inflammation with the use of glucocorticoids and treating MDS-related anemia with the use of erythropoietin.

Our patient overall fits the distinctive clinical profile of VEXAS syndrome as he is male, his age of onset is within the fifth to seventh decade, had macrocytic anemia and systemic inflammatory features.^[10,11]^

VEXAS was first described by Beck and colleagues from the National Institutes of Health in December 2020.^[8]^ It stands for the acronym **V**acuoles, **E**1 enzyme, **X**-linked, **A**utoinflammatory, **S**omatic, and has linked autoimmunity and myelodysplasia to a single genetic lesion. Clinically, fever, inflammatory manifestations, and vacuoles in hematopoietic precursor cells are the main characteristics of this syndrome and a definite diagnosis is made by sequencing the UBA1 gene.^[9]^

UBA1 is linked to cell cycle regulation, signal transduction, apoptosis, DNA damage repair, and transcriptional regulation. Additionally, UBA1 participates in ubiquitination and the neural precursor cell expressed developmentally down-regulated protein 8 pathway for protein folding and degradation, among many other biological processes thus implicating it in mitigating the depletion of ubiquitin during stress.^[12]^ UBA1 appears to be an important regulator of cellular protein homeostasis and has been implicated in neurodegenerative diseases and cancers.^[13]^ As it is characterized by multiple functions, heterogeneity in clinical aspects when mutated is anticipated.^[11]^

Although different studies in the literature have estimated that 10% to 30% of patients with MDS present with concomitant inflammatory and autoimmune features,^[13]^ it is interesting that VEXAS sheds light on the pathophysiological mechanism that links the 2 entities. It is a somatic mutation in the UBA1 gene that alters the cascade involved in the initiation of protein ubiquitylation.^[14]^ As E1 has a multifunctional role in inflammatory signaling, altering its action causes a biological inflammatory profile as presented in VEXAS syndrome. Dysregulation of ubiquitylation leads to autoinflammation through disruption of specific signaling networks and induction of generalized stress pathways.^[14]^

On the other hand, somatic mutations in hematopoietic stem cells initially result in clonal hematopoiesis of insignificant potential, while with the accumulation of additional driven mutations bone marrow failure syndromes and myeloid malignancies (among others) emerge.^[15]^ With the introduction of genomic DNA sequencing, hematological malignancies and autoimmune diseases are now better understood in terms of their molecular background.

After the identification of UBA1 gene mutations, we earned the potential to explain the pathophysiological pathway associated with the clinical manifestations of a group of patients with autoinflammation, cytopenia, and dysplastic bone marrow features.

Currently, the therapeutic management of VEXAS includes different options.^[16]^ However, the efficacy of these treatments remains unclear. Immunosuppressive drugs such as corticosteroids and methotrexate, cytokine targeting agents, JAK2 inhibitors, and the hypomethylating agent azacytidine are some of the therapeutic agents used to treat VEXAS syndrome.^[16,17]^ JAK inhibitors are increasingly used in the treatment of inflammatory and autoimmune diseases and appear to be promising agents because they inhibit cytokines, which drive inflammation and prevent myelofibrosis.^[18,19]^ More data regarding treatment effectiveness are needed to improve the quality of life and outcomes of patients with VEXAS.

## 4. Conclusions

VEXAS syndrome is a myeloid driven, adult-onset inflammatory syndrome associated with hematological neoplasms. VEXAS syndrome as a newly identified entity might often be underestimated because its clinical presentation is notably diverse. Although an association between autoimmunity and myelodysplasia exists in the context of MDS diagnosis, clinicians should continue seeking an alternative diagnosis if atypical disease characteristics appear or if treatment response failure is being noticed. Tight cooperation between various specialists such as hematologists, rheumatologists, and general practitioners would contribute to the correct diagnosis. However, the optimal therapy remains uncertain. Currently, new strategies have been proposed, but the treatment decisions should be individualized.

## Author contributions

**Writing – original draft:** Eleni C. Georgiadi.

**Writing – review & editing:** Ioannis Tsourveloudis, Georgios Vatalis, Paraskevi Kotsi.
